# Stethoscope: a friend or an enemy?

**DOI:** 10.1590/S1516-31802002000100004

**Published:** 2002-01-02

**Authors:** Maria Elisa Zuliani Maluf, Andréa Fogli Maldonado, Marcos Eduardo Bercial, Soraya Ayres Pedroso

**Keywords:** Stethoscope, Infections, Bacterial resistance, Estetoscópios, Infecções, Resistência bacteriana

## Abstract

**CONTEXT:**

The stethoscope is a universal tool in the hospital that is in direct contact with many patients and can therefore be a vector in the dissemination of bacterial infections.

**OBJECTIVE:**

To research the presence of bacteria, fungi and yeast on the stethoscope diaphragm and the resistance of bacteria to antimicrobial drugs.

**DESIGN:**

Descriptive, prospective, non-controlled.

**SETTING:**

A tertiary care hospital.

**SAMPLE:**

Samples were taken randomly from 300 stethoscopes employed by medical staff (medical residents, medical students, nurses and nursing school students) and other sectors of the hospital.

**MAIN MEASUREMENTS:**

Three hundred stethoscope diaphragms used in several sectors of the hospital facilities by medical doctors (63 samples), medical residents (54 samples), medical students (106 samples), nursing school students (33 samples) and specific sectors (36 samples) were analyzed. Material was collected randomly. It was collected with the aid of a sterile swab moistened in physiological solution, inoculated into Brain Heart Infusion media and incubated in an oven for 24 to 48 hours. After this period, the samples were inoculated into blood agar, MacConkey agar and Sabouraud media and identified by Gram staining and biochemical assays. An assay to test bacteria sensitivity to antibiotics was also carried out by the Kirby-Bauer method.

**RESULTS:**

Eighty-seven percent of the analyzed stethoscopes were contaminated. Gram-positive cocci, yeasts, fungi and Gram-positive and negative bacilli were isolated. There was no significant association between the most predominant microorganisms and professional category. Staphylococcus aureus, Staphylococcus negative coagulase and Bacillus were significantly more frequent in relation to the presence of more than one microorganism on the stethoscope diaphragm.

**CONCLUSION:**

Stethoscopes presented a high rate of contamination and their use without precautions can spread nosocomial infections.

## RESUMO

**CONTEXTO:**

O estetoscópio é um instrumento de uso universal no ambiente hospitalar que entra em contato direto com muitos pacientes e, portanto, pode servir como vetor na disseminação de infecções bacterianas.

**OBJETIVO:**

Pesquisar a presença de bactérias, fungos e leveduras no diafragma dos estetoscópios e a resistência bacteriana aos antimicrobianos.

**TIPO DE ESTUDO:**

Descritivo, prospectivo, não-controlado.

**LOCAL:**

Hospital de nível terciário.

**AMOSTRA:**

Utilizamos amostras de 300 estetoscópios colhidas ao acaso e procedentes de médicos, residentes, estudantes de Medicina, enfermeiros, estudantes de Enfermagem e outros setores do hospital.

**VARIÁVEIS ESTUDADAS:**

Foram pesquisados 300 diafragmas de estetoscópios, provenientes aleatoriamente dos diversos setores do hospital e procedentes de médicos (63 amostras), residentes (54 amostras), estudantes de Medicina (106 amostras), enfermeiros (8 amostras), estudantes de Enfermagem (33 amostras) e outros setores (36 amostras). O material foi coletado com uma zaragatoa estéril umedecida em solução fisiológica e inoculada em meio de BHI (Brain Heart Infusion) e levado a estufa por 24 a 48 horas. Logo após, as amostras foram semeadas em meios de ágar-sangue, MacConkey e Sabouraud e identificadas utilizando-se o método de Gram e provas bioquímicas. Posteriormente, realizou-se o teste de sensibilidade aos antimicrobianos através do método de Kirby-Bauer.

**RESULTADOS:**

87% dos estetoscópios analisados estavam contaminados. Foram isolados: cocos Gram-positivos, leveduras, fungos, bacilos Gram-positivos e Gram-negativos. Não houve uma associação significante entre os microorganismos mais prevalentes e a categoria profissional. Em relação à presença de mais de um microorganismo no diafragma dos estetoscópios, *Staphylococcus* *aureus*, *Staphylococcus* coagulase negativos e *Bacillus* foram significantemente mais freqüentes.

**CONCLUSÕES:**

Os estetoscópios apresentam um grande índice de contaminação e sua utilização sem as devidas precauções pode disseminar as infecções hospitalares.

## INTRODUCTION

Nosocomial infections in Brazil are the cause of 45,000 deaths per year and imply an indirect cost of U$ 4.8 billion. This high rate is partially due to the critical conditions to be found in hospital structures. Therefore, all endeavors towards reducing this death rate are valid.^1^

The stethoscope is a tool in constant use among health professionals. It is often passed from one professional to another and is always in direct contact with patients. Disinfection of stethoscopes is an issue that has been neglected.^2,3^ The agents most frequently found on stethoscopes are *Staphylococcus* species, among which are included strains resistant to antibiotics.^2,4,5^ As a consequence of the surge in acquired immune deficiency syndrome and the increase in the number of individuals undergoing chemotherapy or immuno-suppression therapy, it is essential that all the sources of infection be blocked.

The aim of this study was to verify the presence of bacteria, fungi and yeasts on stethoscope diaphragms and test their resistance to antimicrobial drugs.

## METHODS

The study was carried out at the Conjunto Hospitalar de Sorocaba, a tertiary care hospital. Samples were taken randomly from 300 stethoscopes employed by medical staff, medical residents, medical students, nurses and nursing school students, and other sectors of the hospital.

The material was collected from the surface of the stethoscope diaphragms, using a swab moistened in physiological solution. No more than 30 minutes afterwards, it was inoculated into Brain Heart Infusion media.

The cultures were incubated at 36 °C, and after 24 or 48 hours, they were inoculated into blood agar media, MacConkey agar and Sabouraud media.

After the isolation of colonies, bacterioscopic and biochemical assays were carried out to identify the specimens. Antibiotic sensitivity assays were carried out using the Kirby-Bauer test. The data were subsequently analyzed using the Chi-Squared Test (χ^2^).

## RESULTS

Of the total of 300 stethoscopes sampled, 87% were contaminated. Among the contaminated stethoscopes, 96% presented more than one microorganism. The microorganisms isolated were the following: *Staphylococcus aureus* (n=176), *Staphylococcus* negative coagulase (n=153), yeasts (n=148), *Sarcina* (n=64), *Bacillus sp* (n=45), *Streptococcus sp* (n=7), *Acinetobacter sp* (n=2), *Pseudomonas putida* (n=1) and *Klebsiella pnemoniae* (n=1).

Table 1 shows that there was no significant association between the most predominant microorganisms and the professional category, or whether the user was under training or not, or the specific hospital sector.

**Table 1:** Frequency of *Staphylococcus* aureus and *Staphylococcus* negative coagulase according to either professional category or training status

*Groups (n = 176)*	*S. aureus* *present*	*% presence coagulase*	*S. negative* *(n = 153) present*	*% presence*
*Medical Staff*	*45*	*714*	*39*	*620*
*Medical Residents*	*35*	*648*	*29*	*53.7*
*Medical School Students*	*19*	*55.6*	*52*	*490*
*Nurses*	*2*	*250*	*7*	*87.5*
*Nursing School students*	*14*	*424*	*12*	*36.3*
*Others*	*21*	*58.3*	*14*	*38.9*

On the other hand, there was a significant difference between the several agents studied in relation to the presence of more than one microorganism on the stethoscope diaphragm. The *Staphylococcus aureus*, *Staphylococcus* negative coagulase and *Bacillus* were significantly more frequent (P < 0.0001).

Among the *Streptococcus* species, 2 cases of *Enterococcus* and 4 of *Streptococcus viridans* were found. Other microorganisms isolated in lower numbers but of great clinic importance were: *Pseudomonas putida, Klebsiella pneumoniae* and *Acinectobacter sp*.

Table 2 shows the sensitivity of microorganisms to the most frequently used antibiotics in clinical practice.

**Table 2:** Sensitivity of the most frequent agents isolated from stethoscopes to antimicrobial substances - CHS - Sorocaba/SP

	*S. aureus* *(n = 176)*		*S. negative coagulase* *(n = 153)*		*Streptococcus sp* *(n = 7)*	
	*n*	*%*	*n*	*%*	*n*	*%*
*Amikacin*	*144*	*820*	*135*	*880*	*1*	*143*
*Ampicillin*	*11*	*6.5*	*23*	*150*	*0*	*0*
*Carbenicillin*	*28*	*160*	*46*	*300*	*3*	*430*
*Chloramphenicol*	*122*	*69.1*	*111*	*72.5*	*5*	*71.5*
*Cotrimoxazole*	*98*	*55.5*	*93*	*608*	*4*	*570*
*Gentamicin*	*137*	*77.8*	*130*	*850*	*5*	*71.5*
*Netilmicin*	*165*	*93.7*	*145*	*950*	*4*	*570*
*Tetracycline*	*95*	*540*	*84*	*550*	*4*	*570*
*Tobramycin*	*95*	*540*	*102*	*66.5*	*0*	*0*
*Penicillin*	*10*	*56*	*23*	*150*	*1*	*143*
*Erythromycin*	*58*	*330*	*56*	*36.5*	*2*	*286*
*Clindamycin*	*132*	*750*	*117*	*76.5*	*2*	*286*
*Cephalothin*	*157*	*890*	*141*	*920*	*3*	*430*
*Oxacillin*	*94*	*530*	*85*	*550*	*1*	*143*
*Vancomycin*	*176*	*100*	*153*	*100*	*7*	*100*

***Note: The rate of S. aureus resistance to Oxacillin is probably due to the temperature employed during incubation (36 °C).***

## DISCUSSION

*Staphylococcus aureus* was one of the first among the pathogens of human beings to become adapted to the development of antimicrobial substances.

The selection pressure applied to microorganisms for them to become resistant to antimicrobial substances is a consequence of several factors. It comes not only from the use of such drugs in therapeutic or prophylactic applications in Medicine and Dentistry, but also from their use for Veterinary purposes, in food preservation, in the battle against biological elements that are hazardous to mankind, and in the process of livestock-raising. In addition, the free availability of these drugs in drugstores and pharmacies is instrumental.^7^

*Acinectobacter sp* is currently the most common pathogen associated with multiple resistance to antibiotics in hospital infections, especially in Intensive Care Units. Nevertheless, its prevalence in this study was of little significance, with the presence of only one strain.

## CONCLUSION

The stethoscopes presented a high rate of contamination and because of their universal use among health professionals, they can be potential vectors in the dissemination of hospital infections.

It is advisable to regularly clean the diaphragm of the instrument and its parts, in every detail, using a 70% alcohol solution to remove the accumulated organic substances.^6^
